# Structural basis of specific inhibition of tissue-type plasminogen activator by plasminogen activators inhibitor-1

**DOI:** 10.1016/j.dib.2015.12.050

**Published:** 2016-01-06

**Authors:** Lihu Gong, Min Liu, Tu Zeng, Xiaoli Shi, Cai Yuan, Peter A. Andreasen, Mingdong Huang

**Affiliations:** a State Key Laboratory of Structural Chemistry, Danish-Chinese Centre for Proteases and Cancer, Fujian Institute of Research on the Structure of Matter, Chinese Academy of Sciences, Fuzhou, Fujian 350002, China; b University of Chinese Academy of Sciences, Beijing 100049, China; c Danish-Chinese Centre for Proteases and Cancer, Department of Molecular Biology and Genetics, Aarhus University, 8000 Aarhus C, Denmark

**Keywords:** Tpa, Serine protease, PAI-1, Serpin, Michaëlis complex, Crystal structure, Fibrinolysis, Thrombolytic agents, Structural biology

## Abstract

Thrombosis is a leading cause of death worldwide [1]. Recombinant tissue-type plasminogen activator (tPA) is the FDA-approved thrombolytic drug for ischemic strokes, myocardial infarction and pulmonary embolism. tPA is a multi-domain serine protease of the trypsin-family [2] and catalyses the critical step in fibrinolysis [3], converting the zymogen plasminogen to the active serine protease plasmin, which degrades the fibrin network of thrombi and blood clots. tPA is rapidly inactivated by endogenous plasminogen activators inhibitor-1 (PAI-1) [4] (Fig. 1). Engineering on tPA to reduce its inhibition by PAI-1 without compromising its thrombolytic effect is a continuous effort [5]. Tenecteplase (TNK-tPA) is a newer generation of tPA variant showing slower inhibition by PAI-1 [6]. Extensive studies to understand the molecular interactions between tPA and PAI-1 have been carried out [7], [8], [9], [10], [11], [12], [13], [14], [15], [16], [17], [18], however, the precise details at atomic resolution remain unknown. We report the crystal structure of tPA·PAI-1 complex here. The methods required to achieve these data include: (1) recombinant expression and purification of a PAI-1 variant (14-1B) containing four mutations (N150H, K154T, Q319L, and M354I), and a tPA serine protease domain (tPA-SPD) variant with three mutations (C122A, N173Q, and S195A, in the chymotrypsin numbering) [19]; (2) formation of a tPA-SPD·PAI-1 Michaëlis complex in vitro [19]; and (3) solving the three-dimensional structure for this complex by X-ray crystallography [deposited in the PDB database as 5BRR]. The data explain the specificity of PAI-1 for tPA and uPA [19], [20], and provide structural basis to design newer generation of PAI-1-resistant tPA variants as thrombolytic agents [19].

## **Specifications table**

**Table:** 

Subject area	*Biology*
More specific subject area	*Protein structure and biochemistry*
Type of data	*X-ray crystal structure, Mass spectrometry*
How data was acquired	*X-ray diffraction data were collected at Shanghai Synchrotron Radiation Facility. Mass spectra of MALDI-TOF-MS were obtained on a Bruker REFLEX III MALDI-TOF-MS (Bruker-Franzen, Bremen, Germany).*
Data format	*Processed*
Experimental factors	*Recombinant proteins were purified to high homogeneity before use.*
Experimental features	*The structure of the tPA*·*PAI-1 complex was determined by X-ray crystallography.*
Data source location	*City, Country and/or Latitude & Longitude (& GPS coordinates) for collected samples/data if applicable*
Data accessibility	The data is available from the related publication by Gong et al. (http://www.ncbi.nlm.nih.gov/pubmed/26324706), and the structure deposited in the Protein Data Bank (entry 5BRR).

## **Value of the data**

• Determines the crystal structure of the Michaëlis complex between tPA and PAI-1.
• Provides insight on the specificity of PAI-1 for tPA and uPA.
• Identifies key residues of tPA for binding to PAI-1.
• Explains the PAI-1-resisting property of Tenecteplase.
• Offers important clues to design newer generation of PAI-1-resistant tPA variants.

## 1 Data, experimental design, materials and methods

### 1.1 Data and experimental design

We have determined the structure of tPA·PAI-1 Michaëlis complex and identified key residues of tPA for binding to PAI-1 by X-ray crystallography, and the data are summarized in the original publication [19].

We expressed the recombinant PAI-1 variant 14-1B (N150H, K154T, Q319L, and M354I) [21], using the expression vector pT7-PL and BL21 cells as soluble protein [22]. The choice of this particular variant is to obtain PAI-1 in active form, advantageous for crystallization, because the wild type PAI-1 has a half life of only 2 h and has propensity to spontaneously convert into an inactive, so-called latent form, and to aggregate at high concentration [23], [24].

**Fig. 1:**
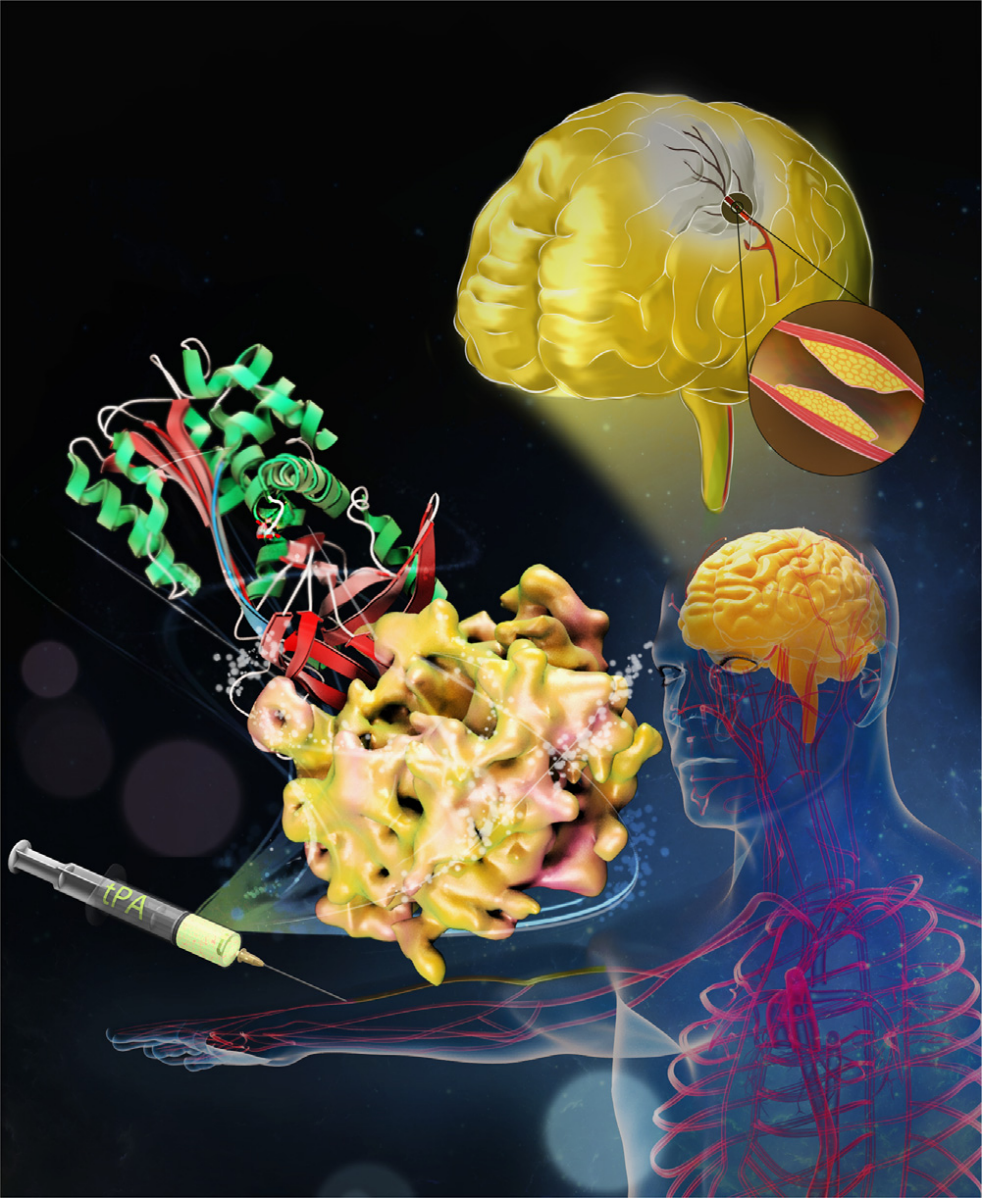
A structural basis to design newer thrombolytics. Recombinant tPA (surface) is the FDA-approved thrombolytic drug. High dose of recombinant tPA is typically needed to lyse clot in stroke patients, partly due to its rapid inactivation by endogenous inhibitor (PAI-1, in ribbon). Such high dosage leads to dangerous side effects, including intracranial hemorrhage and neurotoxicity. Here, the crystal structure of tPA•PAI-1 Michaëlis complex was determined. This structure offers important clues to design newer generation of tPA thrombolytics with reduced PAI-1 inactivation.

PAI-1 inhibits tPA by a suicide-substrate mechanism common to all SERPIN members [23], [25] – see Fig. 1A in the original publication [19]. In this SERPIN mechanism, a long flexible loop of PAI-1 (reaction center loop, or RCL) inserts into the active site of tPA to form a transient Michaëlis complex. The RCL is cleaved by tPA through the classical serine proteolytic mechanism. tPA forms a covalent acyl-enzyme intermediate with PAI-1 by cleaving the scissible bond of PAI-1 RCL, following the Michaëlis complex. Before the hydrolysis of this acyl-enzyme intermediate, the PAI-1 RCL undergoes major conformational changes and inserts itself into the PAI-1 β-sheet A. At the same time, the tPA in the intermediate is pulled to the other side of PAI-1, distorted, and deactivated before the hydrolysis of the acyl-enzyme intermediate can take place.

Human tPA contains a fibronectin type II domain (amino acids 1–50), a growth factor domain (amino acids 51–91), two kringle domains (amino acids 92–261), an interdomain linker (amino acids 262–275) and a serine protease domain (SPD, amino acids 276–527) [2] – see Fig. 1B in the original publication [19]. The tPA-SPD is the catalytic domain responsible for the plasminogen activation and is inhibited by PAI-1. Thus, we used only the recombinant tPA-SPD domain to form the Michaëlis complex with PAI-1. We generated three mutations in tPA-SPD: S478A (or S195A in the chymotrypsin numbering) to render the tPA-SPD catalytically inactive, so the Michaëlis complex does not proceed to a stable, covalent complex; N448Q (or N173Q in the chymotrypsin numbering) to remove the glycosylation on tPA-SPD, increasing the homogeneity of the recombinant protein and facilitating protein crystallization; and C395A (or C122A in the chymotrypsin numbering that will be used throughout the rest of text) mutation to remove the disulfide bond linked to K2 domain – see Fig. 1B in the original publication [19]. The recombinant tPA-SPD mutant was expressed in *P. pastoris* and confirmed by SDS-PAGE and mass spectrometry after trypsin digestion (Table 1).

The recombinant PAI-1 14-1B and tPA-SPD were respectively dialysed into a high-concentration salt (1 M NaCl) and low pH (20 mM Mes pH 6.1) buffer before the Michaëlis complex formation. This condition is required to stabilize PAI-1 at its active form. Subsequently, these two proteins in high salt concentrations and low pH buffer were mixed in a 1:1 M ratio, followed by a dialysis into a low-concentration salt (150 mM NaCl) and neutral pH (20 mM Tris–HCl pH 7.4) buffer. This dialysing step ensures the complex formation similar to that in physiologic condition. A further gel filtration chromatography purification yielded a complex of greater than 99% purity.

## 2 Materials and methods

### 2.1 Recombinant protein production

The recombinant PAI-1 mutant 14-1B [21] containing four point mutations (N150H, K154T, Q319L, and M354I), and a hexa-His-tag was expressed in *E. coli*, using the expression vector pT7-PL and BL21 cells as previously described [22]. The recombinant tPA-SPD was expressed in *P. pastoris* X-33. This strain facilitates the formation of five disulfide bonds (C42-C58, C50-C111, C100-C182, C136-C201, C168-C182 in chymotrypsin numbering) in tPA-SPD with a high yield about 50 mg recombinant protein per liter medium – see in the original publication [19].

### 2.2 The peptide mass fingerprinting of tPA-SPD by MALDI-TOF mass spectrometry

The SDS-PAGE was performed using 15% polyacrylamide gels. Following SDS-PAGE, the gels were stained with 0.1% (w/v) Coomassie brilliant blue R-250 in 25% (v/v) ethanol and 10% (v/v) acetic acid. The gel digestion was performed using a modified version of previously published protocol [26]. Briefly, the gel band containing 100 ng tPA-SPD was excised from the 15% two-dimensional SDS-PAGE gel, cut in pieces, and destained by washing with 50% (v/v) acetonitrile in 100 μl of 25 mM NH_4_HCO_3_ for 30 min at room temperature. The gel pieces were then dried in a SpeedVac Vacuum (Savant Instruments, Holbrook, NY, USA) and rehydrated at 4 °C for 15 min in 3–5 μl digestion solution (25 mM NH_4_HCO_3_ and 12.5 ng/μl modified sequence-grade trypsin). Then 3–5 μl of digestion solution without trypsin was added to keep the gel pieces wet during the digestion. After overnight incubation at 37 °C, the digestion was stopped with 5% trifluoroacetic acid (TFA) for 20 min. The peptides were extracted by 20 μl of 5% TFA for 1 h at 37 °C and then by 20 μl of 2.5% TFA/50% acetonitrile for 1 h at 37 °C. The combined supernatants were evaporated in the SpeedVac Vacuum and dissolved in 4 μl 0.5% aqueous TFA for MS analysis.

All mass spectra of MALDI-TOF-MS were obtained on a Bruker REFLEX III MALDI-TOF-MS (Bruker–Franzen, Bremen, Germany) in positive ion mode at an accelerating voltage of 20 kV with the matrix of α-cyano-4-hydroxy cinnamic acid. The spectra were internally calibrated using trypsin autolysis products. The peptide mass fingerprinting obtained was used to search through the SWISS-PROT and NCBI database by the Mascot search engine (http://mascot.proteomics.com.cn/) with a tolerance of ~+0.3 D and one missed cleavage site.

### 2.3 X-ray crystallography

The tPA-SPD·PAI-1 Michaëlis complex was formed by mixing tPA-SPD and PAI-1 in a 1:1 M ratio at low concentration (~ 0.5 mg/ml), followed by dialysis into 20 mM Tris–HCl pH 7.4, and 150 mM NaCl, concentration to 0.5 ml volume for a further gel filtration chromatography purification, which yielded to a complex of greater than 99% purity. The purified complex was then concentrated to 10 mg/mL before setting up crystallization trials. Crystals of the tPA-SPD·PAI-1 Michaëlis complex were grown at 20 °C with the sitting drop method by mixing equal volumes of protein solution and precipitant solution (8% PEG-6000 and 0.1 M Tris pH 7.4), and appeared quickly within one day. However, the crystals always appeared as very thin plates, and decayed rapidly in the X-ray beam, posing great difficulty for X-ray data collection. Most crystals diffracted to only 4–5 Å at Shanghai Synchrotron Radiation Facility (SSRF) BL-17U beam line, and the diffracting spots often appeared as elongated or splitted shapes. After many crystallization and data collection trials for one and half years, one 3.16 Å data set was finally obtained at SSRF beam line BL17U using 25% glycerol as cryoprotectant at a wavelength of 0.979 Å. The data were processed and scaled using the HKL2000 program package [27]. The crystal belongs to P2_1_2_1_2_1_ space group with one complex in the crystallographic asymmetric unit. The structure of the tPA-SPD·PAI-1 Michaëlis complex was solved by molecular replacement method using MolRep program [28], which gave very strong and unambiguous solutions. A tPA-SPD molecule was first positioned inside the crystal lattice using the structure of the tPA-SPD catalytic domain (PDB code 1A5H) [29] as a searching model and all the X-ray data up to 3.3 Å. The molecular replacement using MolRep gave a contrast of 12.33, a signal to sigma ratio for translational function of 16.02, and a correlation coefficient of 0.365. Next, the position of PAI-1 was searched using the model of active stable variant of PAI-1 (Protein Data Bank code 1DVM) [30] while fixing the already positioned tPA-SPD model, giving only one translational function with a signal to sigma ratio of 19.4, and a correlation coefficient of 0.538. The molecular replacement model was subjected to iterative refinement and manual model rebuilding using Refmac [31] and Coot [32], respectively, giving a final R factor and R_free_ factor of 0.20 and 0.27, respectively. The structure was validated with PROCHECK [33] and analyzed by PyMOL [34] and PISA [35]. The final refined crystal structure of tPA-SPD·PAI-1 Michaëlis complex was deposited in PDB with the code *5BRR*.

## Figures and Tables

**Table 1:** Trypsin digested fragments of recombinant tPA-SPD SPD from MALDI-TOF-MS and the expected fragment mass.

Mr observed (Da)	Mr calculated (Da)	Peptide sequence
1387.1	1386.8004	^53^FPPHHLTVILGR^64^
1335.8	1335.6328	^142^HEALSPFYSER^152^
1179.4	1179.6157	^239^VTNYLDWIR^247^
878.8	878.4618	^231^DVPGVYTK^238^
722.4	722.3831	^160^LYPSSR^165^
