# Supplementary material for: Structural basis of specific inhibition of tissue-type plasminogen activator by plasminogen activators inhibitor-1
Source: Data Brief. 2016 Jan 6;6:550–5. doi: 10.1016/j.dib.2015.12.050 (PMC4731420; doi:10.1016/j.dib.2015.12.050)
Supplement: Supplementary file 1 — Supplementary material [file mmc1.docx]

Structural basis of specific inhibition of Tissue-type Plasminogen Activator by Plasminogen Activators Inhibitor-1

**Authors:** Lihu Gong^1,2^, Min Liu^1,2^, Tu Zeng^1^, Xiaoli Shi^1^, Cai Yuan^1^, Peter A. Andreasen^3^, Mingdong Huang^1,2,^^*^

**Affiliations:** ^1^State Key Laboratory of Structural Chemistry, Danish-Chinese Centre for Proteases and Cancer, Fujian Institute of Research on the Structure of Matter, Chinese Academy of Sciences, Fuzhou, Fujian, China

^2^University of Chinese Academy of Sciences, Beijing, 100049, China

^3^Danish-Chinese Centre for Proteases and Cancer, Department of Molecular Biology and Genetics, Aarhus University, Denmark

^*^**Contact email:** [mhuang@fjirsm.ac.cn](mailto:mhuang@fjirsm.ac.cn)

**Conflicts of interest: none.**
